# In Vivo Evaluation of Immune-Enhancing Activity of Red Gamju Fermented by Probiotic *Levilactobacillus brevis* KU15154 in Mice

**DOI:** 10.3390/foods10020253

**Published:** 2021-01-26

**Authors:** Eunju Park, Kee-Tae Kim, Mijoo Choi, Yunjung Lee, Hyun-Dong Paik

**Affiliations:** 1 Department of Food Nutrition, Kyungnam University, Changwon 51767, Korea; pej@kyungnam.ac.kr (E.P.); orange0859@nate.com (M.C.); hjlee@kyungnam.ac.kr (Y.L.); 2 Department of Food Science and Biotechnology of Animal Resources, Konkuk University, Seoul 05029, Korea; richard44@hanmail.net

**Keywords:** *Levilactobacillus brevis*, immune-enhancing activity, gamju, lactic acid bacteria, cytokine

## Abstract

The purpose of this study was to evaluate the immune-enhancing effect of red gamju fermented with *Levilactobacillus brevis* KU15154, isolated from kimchi, as a biofunctional beverage using mice. Thirty-two mice were used, and after a 2-week feeding, the growth, cytokine and immunoglobulin production, and immune-related cell activation (phagocytes and natural killer [NK] cells) of the mice were evaluated. The red gamju- (SR) and fermented red gamju- (FSR) treated groups had 3.5–4.0-fold greater T-cell proliferation ability than the negative control group. IFN-γ production in the FSR group (15.5 ± 1.2 mg/mL) was significantly higher (*p* < 0.05) than that in the SR group (12.5 ± 1.8 mg/mL). The FSR group (502.6 ± 25.8 μg/mL) also showed higher IgG production levels than the SR group (412.2 ± 44.8 μg/mL). The activity of NK cells treated with FSR was also greater than that of cells treated with SR but it was not significant (*p* ≤ 0.05). Further, the phagocytic activity of peritoneal macrophages was higher in both SR and FSR groups than in the control group but was not significantly different (*p* < 0.05) between the SR and FSR groups. In conclusion, *L. brevis* KU15154 may be applied in the fermentation of bioactive food products, such as beverages or pharmaceutical industries, to potentially improve immunity.

## 1. Introduction

The “immunity-enhancing activity” became one of the biggest needs for modern generations due to their increasing susceptibility to harmful factors such as new pathogens and virus, stress, and other unknown bionic substances. Therefore, various functional food products with immune-enhancing effects have been increasingly in-demand worldwide.

Recently, many people have become increasingly interested in functional foods that contain natural ingredients to maintain their health or prevent diseases [1,2] and many food scientists have eagerly studied bio-functional materials such as probiotics, phytochemicals, organic materials produced by microorganisms (fermentation or bioconversion). In particular, lactic acid bacteria (LAB) are considered the most commonly used biomaterials, as studies have shown that the LAB-mediated treatment has many benefits in illnesses, such as foodborne diseases, inflammation, intestinal diseases, and cancer [3,4,5,6]. Many kinds of LAB can be isolated from kimchi, which is a representative traditional Korean food manufactured by naturally fermenting the vegetables such as cabbage and radish at a low temperature. Many kinds of LAB strains including *Lactobacillus* spp., *Lactococcus* spp., *Leuconostoc* spp., and *Pediococcus* spp. have been isolated from kimchi [7,8,9].

Cereals contain a large amount of dietary fibers and supply bioactive compounds including various vitamins and minerals [10]. In particular, some researchers have shown that fermenting cereal products with LAB increases their digestibility and sensory characteristics, with improved biofunctionality and storage periods [9]. Gamju is one of the traditional beverages in Korea as a dessert or sports drink and is made with rice. The major ingredients of gamju are steamed rice, malt juice (wort), and water. Hydrolysis enzymes in wort decompose the rice starch into maltose or glucose, and the taste of the final product is sweet [11,12]. *Monascus anka*, called “red mold”, has been used as a type of seed culture, called “koji”, for saccharification in alcoholic beverages and as a traditional fermented seasoning in Asia, since this strain can hydrolyze the starch in rice into mono- or disaccharides using saccharolytic enzymes [13,14]. Furthermore, it can improve the flavor of malt juice during gamju processing due to its sweetness, this gamju is called “red gamju.” Nowadays, cereal-based beverages, such as gamju, have been studied to improve their physicochemical and sensory characteristics by adding other ingredients, such as elm root extract [15] or pumpkin paste [13]. However, only a few studies on quality improvement was done by making changes in the processes, such as fermentation [16].

The aim of this study was to evaluate the in vivo immune-enhancing activity of gamju, fermented by the novel LAB strain isolated from kimchi as a functional food material to improve its biofunctional activity, using mice. These results may promote the development of biofunctional cereal-based beverages with an immune-enhancing activity in the food industry.

## 2. Materials and Methods 

### 2.1. Isolation and Identification of Strains and Tissue Culturing 

The lactic acid bacteria (LAB) strains used in this study were screened from radish-based kimchi. A 1 g of each food sample was serially diluted and spread on de Man, Rogosa, and Sharpe (MRS) agar (BD Biosciences, Franklin Lakes, NJ, USA) for incubation at 37 °C for 24 h. Then, every colony was isolated and incubated anaerobically under the same culture conditions. Potential probiotic LAB strains were identified as *Levilactobacillus brevis* using the 16S rRNA sequencing technique by Bionics Inc. (Seoul, Korea). The analysis of sequencing were performed using the basic local alignment search tool (BLAST) website (http://blast.ncbi.nlm.nih.gov) and by comparison with the GENBANK database. This strain was named as *Levilactobacillus brevis* KU15154.

Yac-1 cells in mouse lymphoma were cultured in RPMI 1640 (Hyclone, Logan, UT, USA) supplemented with 10% FBS (Gibco BRL, Grand Island, NY, USA) and 1% penicillin/streptomycin (Gibco BRL, Grand Island, NY, USA) and were maintained in a 75T flask (Falcon, Becton Dickinson, NJ, USA) at 37 °C under a humidified atmosphere of 5% CO_2_.

### 2.2. Chemicals and Sample

The Roswell Park Memorial Institute (RPMI) 1640 media and fetal bovine serum (FBS) were purchased from Hyclone (Logan, UT, USA) and penicillin was obtained from Gibco BRL (Grand Island, NY, USA). Lipopolysaccharide (LPS) and concanavalin A (Con A) were purchased from Sigma-Aldrich Chemical Co. (St Louis, MO, USA). The immunoglobulin (Ig)A, IgE, and IgG ELISA assay kits were purchased from the Immunology Consultants Laboratory (Newberg, OR, USA). IL-2, IL-4, IL-6, IL-10, IFN-γ, and TNF-α ELISA assay kits were purchased from the BD DuoSet ELISA set kit (BD Biosciences, San Diego, CA, USA). The natural killer (Nk) cell activity was purchased from the Cytotox 96^®^ non-radioactive cytotoxicity assay kit (Promega, Madison, USA). The CytoSelect^TM^ 96-well phagocytosis assay (zymosan substrate) kit was purchased from Cell Biolabs, Inc., (San Diego, CA, USA). The trizol reagent and QuantiSpeed SYBR No-Rox kit were taken, respectively, from Invitrogen (Carlsbad, CA, USA) and Philekorea (Seoul, Korea).

The red gamju was manufactured and fermented using the modified methods of Yang et al. [16]. The red gamju was prepared as follows: Rice (10 kg) was soaked in tap water for 2 h and cooked for 40 min. Then, the cooked rice was cooled to 35 °C and inoculated with 0.2% (*w*/*w*) koji. In this study, koji was manufactured by the inoculation of *M. anka* spores on cooked rice and by incubation at 30 °C for 4 days. To produce the red gamju, cooked rice, koji, and distilled water were blended at a ratio of 1:1:4 (by weight). Saccharification of a mixture was performed by incubating in a water bath at 60 °C. The saccharified red gamju was heat-treated at 121 °C for 15 min for sterilization. After cooling, the fermentation was performed by the inoculation of *L. brevis* KU15154 at 10^5^ CFU/mL of gamju and by incubation anaerobically at 37 °C for 24 h.

### 2.3. Experimental Animal and Treatment

The animal experiment for this study was approved by the Kyungnam University Instrumental Animal Care and Use Committee (Changwon, South Korea) (KUIAC-18-03). Four-week-old male BALB/c mice were purchased from Koatech Animal Inc. (Pyeongtaek, South Korea) and were brought up in a ventilated room with a 12-h day-night cycle at 25 ± 2 °C. The relative humidity was controlled to 50 ± 5%. Experimental animals were acclimated for 1 week before starting the experiment and were provided with a standard pellet chow and freshwater *ad libitum*. In addition, swimming as a high intensity exercise was used to depress the immune activity by stress. The swimming of mice is performed in a water pool at 34 °C until exhausted. A total of 32 mice (all males) were randomly assigned to four groups with eight animals per group: Negative control (NC, no swimming and no supplement), positive control (PC, swimming and no supplement), SR (swimming and supplemented with freeze-dried red gamju powder; 19.5 g/kg body weight), and FSR (swimming and supplemented with freeze-dried fermented red gamju powder; 18.5 g/kg body weight). SR and FSR concentrations were calculated by applying an extrapolation method based on the daily intake of drinks of adult Koreans (Korean Statistical Information Service, http://kosis.kr/statHtml/statHtml.do?orgId=117&tblId=DT_11702_N021&vw_cd=MT_ZTITLE&list_id=117_11702_A01_033&seqNo=&lang_mode=ko&language=kor&obj_var_id=&itm_id=&conn_path=MT_ZTITLE [accessed 2016]), and oral administration was performed for 2 weeks using jonde at the same time every day. The weight and dietary intake were measured at a certain time once a week, and the average swimming time for 2 weeks was considered the swimming time before dissection. To obtain sufficient macrophages, the mice were intraperitoneally injected with 2 mL of a 4% thioglycollate medium (Sigma-Aldrich Chemical Co., St Louis, MO, USA). Three days later, the mice were sacrificed by cervical dislocation. One of the extracted spleens was stored at –80 °C for RT-PCR, while the other one was aseptically isolated and crushed by passage through a sterile plastic strainer. The separated splenocytes and macrophages were seeded in 96-well microplates at an initial concentration of 1 × 10^6^ cells/well and incubated for 12 h.

### 2.4. T- and B-Cell Proliferation

The splenocytes were treated with RPMI media, LPS (1 μg/mL), or Con A (1 μg/mL). T- and B-cell proliferation assays (EZ-CyTox, Daeil Lab Service, Seoul, Korea) were performed using Con A or LPS, as described by Park et al. [17].

### 2.5. Cytokine Production

The cytokines of splenocytes treated with RPMI, LPS (1 μg/mL), or Con A (1 μg/mL) were determined using the BD DuoSet ELISA set kit (BD Biosciences, San Diego, USA). In this study, IL-2 and IFN-γ were tested as Th1 type cytokines and IL-4, IL-6, IL-10, and TNF-α were tested as Th2 type cytokines. All the experiments were conducted according to the manufacturer’s manuals.

### 2.6. Real-Time Quantitative PCR (RT-PCR)

Total RNA was extracted from the spleen using the Trizol reagent according to the manufacturer’s protocol. Reverse transcription was performed using the Superscript First-Stand cDNA Synthesis kit. The sequences of primers for amplification are presented in Table 1. A thermal cycling condition for PCR reactions was set to 95 °C for 10 min, followed by 55 cycles of 95 °C for 15 s, 55 °C for 20 s, and 72 °C for 20 s. RT-PCR reactions were carried out with an iCycler real-time PCR machine (Biorad, Hercules, CA, USA) using the QuantiSpeed SYBR No-Rox kit. The mRNA levels of each gene were normalized against those of GAPDH.

### 2.7. Serum Immunoglobulins (Ig) Production

Blood was obtained from the ophthalmic artery, and the serum was separated by centrifugation at 220× *g* rpm for 10 min. Serum IgA, IgE, and IgG levels were determined using ELISA kits according to the manufacturer’s manuals.

### 2.8. Natural Killer (NK) Cell Activity

Splenocytes isolated from the spleen were cultured in 96-well plates at a density of 1 × 10^5^ cells/well, and co-cultured with 1 × 10^4^ Yac-1 cells/well. The ratio of effector cells to target cells was 10:1, and the plates were incubated at 37 °C in a 5% CO_2_ incubator for 4 h. After incubation, 50 μL of the supernatant was dispensed into a new 96-well plate using the Cytotox 96^®^ Non-Radioactive Cytotoxicity Assay kit (Promega). All the experiments were conducted according to the manufacturer’s protocol.

### 2.9. Phagocytic Activity

The CytoSelect 96-well phagocytosis assay (zymosan substrate) kit (Cell Biolabs, Inc., San Diego, CA, USA) was used to evaluate the phagocytic activity of peritoneal macrophages. For the activation of macrophages, the zymosan (Zy) substrate in the kit was used. All the experiments were conducted according to the manufacturer’s protocol.

### 2.10. Statistical Analysis

All the experiments were performed in triplicate experiments. After conducting a one-way analysis of variance using the SPSS statistical program (SPSS Statistics 18.0; SPSS, Inc., Chicago, IL, USA), the significance of the experimental results was analyzed with Duncan’s multiple-range test. 

## 3. Results

### 3.1. Body Weight, Food Intake, and Feed Efficiency Ratio (FER)

The effect of gamju and fermented red gamju on the body weight and FER is shown in Table 2. The daily increase in body weight and FER on the daily diet intake were not significantly different among the groups (*p* < 0.05). This means that fermentation using *L. brevis* KU15154 did not affect the growth and digestibility of mice during the 2-week bleeding. This was in line with the study of Lee et al. [18], where there was no difference in the increased body weight between the control and groups fed with probiotics isolated from kimchi for 8 weeks. In addition, the weight changes of the organs (the liver, kidney, spleen, and heart) were also not affected during the 2-week bleeding. 

### 3.2. Proliferation of T- and B-Cells

**Figure 1:**
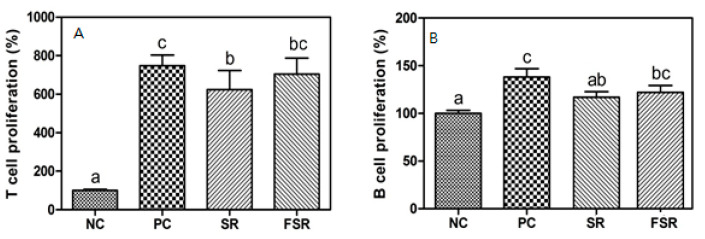
Effects of fermented red gamju on proliferation of T- (**A**) and B- (**B**) cells from splenocytes in Balb/c mice. NC: Negative control group; PC: Positive control group; SR: Sweet red gamju-treated group; FSR: Fermented sweet red gamju-treated group. Alphabets of data were designed according to the difference at *p* < 0.05.

The effects of red gamju fermentation by *L. brevis* KU15154 on the proliferation abilities of T- and B-cells were evaluated and are presented in Figure 1. In the case of T-cells proliferation, the PC (a group treated with ConA), SR, and FSR groups had 3.5–4.0-fold higher cell proliferation ability than the NC (not treated) group, while there was no significant difference in the proliferation ability (*p* < 0.05) between SR and FSR (Figure 1A). 

After the LPS treatment, the proliferation ability of B-cells in PC was significantly greater (*p* < 0.05) than in NC (Figure 1B). Additionally, the proliferation ability of B-cells in the FSR group was lower than in the PC group, but had a similar level with the SR group.

In contrast, the proliferation ability of B-cells in the FSR group was higher than in the SR group, but the difference was not significant (*p* < 0.05). From these results, it was shown that, upon treatment with mitogen, the proliferation of immune-related cells may be stimulated by the intake of fermented gamju. 

### 3.3. Production of Cytokines

**Figure 2:**
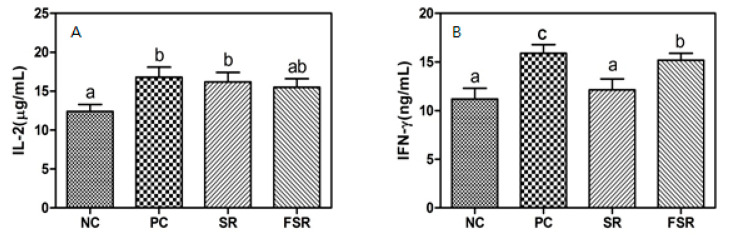
Effects of fermented red gamju on the production of Th1-type cytokines (IL-2 (**A**) and IFN-γ (**B**)) from splenocytes in Balb/c mice. NC: Negative control group; PC: Positive control group; SR: Sweet red gamju-treated group; FSR: Fermented sweet red gamju-treated group. Alphabets of data were designed according to the difference at *p* < 0.05.

As shown in Figure 2, the production of IL-2 and IFN-γ, as Th1-type cytokines, was increased in the PC group than in the NC group. 

In particular, the production of IL-2 in the splenocytes of SR and FSR groups was greater than in the splenocytes of the NC group, but the difference between the two treated groups was not significant (*p* < 0.05). The FSR group showed a lower IFN-γ production than the PC group, significantly (*p* < 0.05). However, the IFN-γ production in the FSR group (15.5 ± 1.2 mg/mL) was significantly higher than in the SR group (12.5 ± 1.8 mg/mL). 

**Figure 3:**
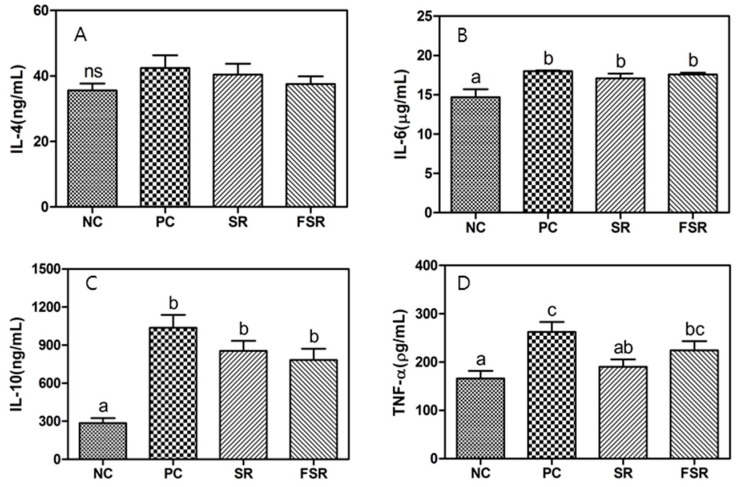
Effects of fermented red gamju on the production of Th2-type cytokines (IL-4 (**A**), IL-6 (**B**), IL-10 (**C**), and TNF-α (**D**)) from splenocytes in Balb/c mice. LPS: Lipopolysaccharide; NC: Negative control group; PC: Positive control group; SR: Sweet red gamju-treated group; FSR: Fermented sweet red gamju-treated group. Alphabets of data were designed according to the difference at *p* < 0.05.

The production of IL-4, a Th2-type cytokine, was determined, as shown in Figure 3. The production of IL-6, IL-10, and TNF-α in the PC group was higher than in the NC group, but the IL-4 production was not significantly different (*p* < 0.05). The TNF-α production was higher in the FSR group than in the SR and NC groups. Additionally, the production of IL-6 and IL-10 cytokines did not differ significantly (*p* < 0.05) between the SR and FSR groups. 

Table 3 shows the genetic expression of IL-4, IL-6, and Il-10 in the fermentation of sweet red gamju using RT-PCR. RT-PCR revealed that IL-4, IL-6, and IL-10 mRNA expression levels significantly decreased to a greater extent in the PC group than in the NC group (*p* > 0.05), and that the SR and FSR groups had higher cytokine mRNA levels than the PC group. In particular, the intake of FSR increased the mRNA expression of IL-6 and IL-10 (*p* < 0.05).

### 3.4. Production of Ig

Table 4 shows the Ig concentrations in the serum of mice after feeding with SR and FSR. The results showed that IgG concentrations in the PC group were higher than in the NC group. IgG concentrations in both SR and FSR groups were significantly higher (*p* < 0.05) than those in the NC group. The FSR group (502.6 ± 25.8 μg/mL) had a higher IgG production than the SR group (412.2 ± 44.8 μg/mL). This means that both groups regulated and prevented the overproduction of Ig. The concentrations of IgA and IgE did not differ significantly (*p* < 0.05) between the treatment groups.

### 3.5. Activation of NK Cells against Yac-1 Cells

**Figure 4:**
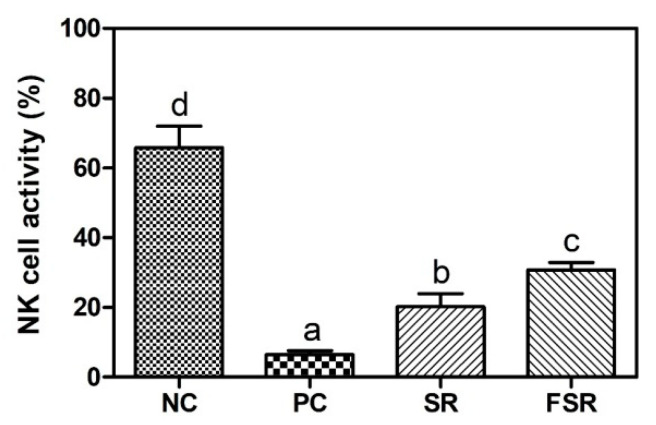
Effects of fermented red gamju on the NK cell activity against Yac-1 from the peritoneal macrophage in Balb/c mice. NC: Negative control group; PC: Positive control group; SR: Sweet red gamju-treated group; FSR: Fermented sweet red gamju-treated group. Alphabets were designed according to the difference at *p* < 0.05.

The activation of NK cells against Yac-1 cells is shown in Figure 4. As a result, the activity of NK cells treated with PC was significantly lower (*p* < 0.05) than that of cells treated with NC, SR, and FSR. In addition, the activity of NK cells treated with FSR was greater than that of cells treated with SR.

### 3.6. Phagocytic Activity

**Figure 5:**
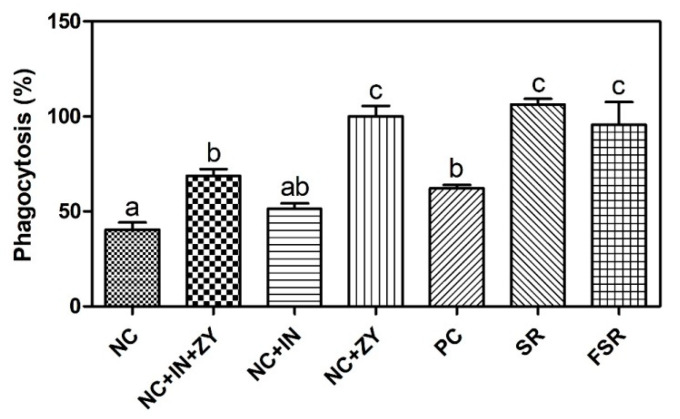
Effects of fermented red gamju on the phagocytic activity of peritoneal macrophage in Balb/c mice. NC: Cell only; NC + ZY + In: NC + zymosan + zymosan inhibitor; NC + In: NC + zymosan inhibitor; NC + ZY: NC + zymosan; PC: Positive control; SR: Sweet red gamju-treated group; FSR: Fermented sweet red gamju-treated group. Alphabets were designed according to the difference at *p* < 0.05.

The activated phagocytes were treated with SR or FSR, and the relative activities in the treated groups were evaluated as shown in Figure 5 (the phagocytic activity in the NC + ZY group was set as 100%). The activities in both SR and FSR groups were higher than in the NC group but were not significantly different (*p* < 0.05) when compared with the NC + ZY group.

## 4. Discussion

The spleen, an organ found in vertebrates, is similar in structure to a large lymph node. The spleen plays an important role in the immune system. Splenocytes are white blood cells in the spleen that consist of a variety of cell populations, such as T- and B- lymphocytes. Splenocyte proliferation has been used as an index of the immune-enhancing activity since lymphocyte differentiation in the spleen is caused by antigens [19].

The effects of red gamju fermentation by *L. brevis* KU15154 on the proliferation abilities of T- and B-cells were evaluated and are presented in Figure 1. In this study, Con A, which is a mannose/glucose-binding plant lectin extracted from the Jack bean, was used as a T-cell mitogen to activate an immune response [20]. The PC, SR, and FSR groups had a higher cell proliferation ability than the non-treated group but there was no significant difference in the proliferation ability between SR and FSR.

LPS was used as the stimulator of mitogen in B-lymphocytes, which induces B-cell diffusion and differentiation. Furthermore, it induces the secretion of cytokines, such as IL-4, IL-6, and IL-10, from B-cells [21,22]. In this study, the proliferation ability of B-cells in the fermented gamju-treated group was lower than in the positive control group, but had a similar level with the negative group.

Cytokines are small proteins (molecular weight: Approximately 25 kD) produced in response to various external stimuli. These molecules are known to stimulate other cells immunologically by binding to specific receptors on other cell surfaces [23]. In general, cytokines can be classified as Th1- and Th2-types. Th1 cytokines increase the biological activities of macrophages and stimulate the phagocytosis, while Th2 cytokines increase the antibody production by stimulating B-cells. As Th1-type cytokines, the production of IL-2 in the splenocytes of both fermented- and non-fermented-gamju treated groups was greater than in the splenocytes of the negative control group. In addition, the IFN-γ production in the fermented-gamju treated group was significantly higher than in the non-fermented red gamju treated group.

In the case of Th2-type cytokines, the production of IL-6, IL-10, and TNF-α in the PC group was higher than in the negative control group. In particular, the TNF-α production of the fermented red gamju-treated group was higher in this study. In addition, the sweet red gamju-treated group had higher cytokine mRNA levels than the positive control group and, particularly, the intake of the fermented sweet red gamju increased the mRNA expression of IL-6 and IL-10 (*p* < 0.05).

A high-intensity exercise such as swimming for a long time depresses the innate immune system including the phagocytic activity and causes the degranulation of neutrophils, NK cell cytotoxicity, as well as an imbalanced production of Th1 and Th2 cytokines [24]. In previous studies, most cytokines are known to decrease after a high-intensity exercise [25]. Some prior studies suggest that cytokine gene expression increases during recovery after a high-intensity exercise and/or no difference in the amount of cytokines in the spleen after a high-intensity exercise [26,27]. Thus, the increase in IL-4 and IL-6 mRNA expansion in the spleen, unlike the cytokine in the blood, is thought to change during recovery after a high-intensity exercise.

Many studies have shown that cytokine induction in vivo is dependent on the concentration and treatment with the lipoteichoic acid (LTA). In particular, Jeong et al. [28] reported that LTAs in LAB induced the production of TNF-α. Therefore, the immune-enhancing activity of this strain may occurr by the interaction of LTA with the splenocytes of mice.

In addition, Th1-type cytokines affect the immune response by activating macrophages and stimulating thrombocytes, while the Th2-type cytokine increases the production of antigens by stimulating B-cells. Many studies suggested that Th1/Th2-type cytokines should be regulated properly for an immunological balance [29,30,31]. In particular, TNF-α, a Th2-type cytokine, is known to be a major cytokine since it induces inflammation and apoptosis [32] and is recognized as a biomarker of stress [33].

Shin et al. [24] have reported that excessive exercise negatively affects the immune activity and that exercising constantly affects the production of Ig. The results of this study showed that IgG concentrations in non-fermented or fermented sweet red gamju-treated groups were significantly higher (*p* < 0.05) than those in the control group. In particular, the fermentation of sweet red gamju SR group increased the IgG production than the SR group. This means that both groups regulated and prevented the overproduction of immunoglobulins. However, the concentrations of IgA and IgE did not differ significantly between the treatment groups. The concentration of blood Ig is a very important clinical factor, as the decline in blood Ig results from abnormal conditions, such as malnutrition or immunosuppression, and its increase results from liver disease, infection, or autoimmune disorder. Therefore, the abnormal amount of blood Ig indicates an immune system disorder, although its homeostasis is different depending on environmental, psychological, and nutritional conditions [34].

In the case of activation of NK cells against Yac-1 cells, it appeared that the activity of NK cells treated with PC was significantly lower (*p* < 0.05) than that of cells treated with any other treated groups. In particular, the activity of NK cells treated with fermented sweet red gamju was greater than that those treated with non-fermented sweet red gamju. NK cells play a role in pathogen elimination [35] and have a tumoricidal activity [36]. Many researchers have shown that probiotics enhance the activity of NK cells [37,38].

The phagocytic activity of macrophages was assayed as an indicator of the immune activity and function activation. Monobe et al. [39] reported that antioxidants stimulate the phagocytic activity. In this study, the activities in both fermented and non-fermented sweet red gamju-treated groups were higher than in the control group. Some researchers have reported that LAB decreases the infection of enteropathogens and increases the cytokine levels in mucosal cells and phagocytic activity [40,41,42,43].

Kim et al. [44] reported that the *Levilactobacillus brevis* cell itself as a probiotic has immuno-enhancing effects. This means that the intake of (fermented) gamju with LAB, such as *L. brevis* KU15154, can be helpful for the immune system. Our research was conducted only in mice and the effect on humans based on these results is necessary for a further practical application.

## 5. Conclusions

A previous study reported that *L. brevis* KU15154 isolated from kimchi showed a high resistance to gastric acid and bile salts, thus demonstrating that it can be used as a probiotic strain. *L. brevis* KU15154 was found to be safe since it does not produce harmful enzymes that negatively affect the health and cause antibiotic resistance. Furthermore, in this study, the fermentation of sweet red gamju with *L. brevis* KU15154 can improve effectively the immune-enhancing activity by increasing the production of some cytokines in mice after a 2-week feeding. Based on the results, *L. brevis* KU15154 may be used as a novel probiotic in the food or pharmaceutical industry to improve human immunity against various diseases.

## Figures and Tables

**Table 1:** Sequences of primers for real-time PCR.

Gene Name	Description	Primer Sequences	
IL-4	Interleukin-4	Forward:	ARCATCGGCATTTTGAACGA
		Reverse:	AAGCCCGAAAGAGTCTCTGC
IL-6	Interleukin-6	Forward:	CCTTCCTACCCCAATTTCCA
		Reverse:	CGCACTAGGTTTGCCGAGTA
IL-10	Interleukin-10	Forward:	TAAGGCTGGCCACACTTGAG
		Reverse:	AGTTTTCAGGGATGAAGCGG
GAPDH	Glyceraldehyde 3-Phosphate dehydrogenase	Forward:	CGCTCTCTGCTCCTCCTGTTC
		Reverse:	CGCCCAATACGACCAAATCCG

**Table 2:** Feed efficiency ratio and organ weight of experimental groups for 2 weeks.

Items	NC ^1^	PC	SR	FSR
Food intake (g/day)	3.13 ± 0.00 ^2,ns^	3.50 ± 0.00	2.98 ± 0.00	3.14 ± 0.00
Weight gain (g/day)	0.05 ± 0.01	0.05 ± 0.02	0.05 ± 0.05	0.07 ± 0.05
FER ^3^	1.52 ± 0.38 ^ns^	1.53 ± 0.51	1.80 ± 0.35	2.11 ± 1.54
Liver (g)	0.82 ± 0.06 ^ns^	0.82 ± 0.05	0.85 ± 0.06	0.85 ± 0.4
Kidney (g)	0.28 ± 0.01	0.29 ± 0.01	0.28 ± 0.01	0.28 ± 0.01
Spleen (g)	0.06 ± 0.00	0.07 ± 0.01	0.06 ± 0.00	0.07 ± 0.00
Heart (g)	0.11 ± 0.01	0.11 ± 0.00	0.10 ± 0.01	0.11 ± 0.00

^1^ FSR: Fermented sweet red gamju-treated group; NC: Negative control group; PC: Positive control group; SR: Sweet red gamju-treated group. ^2^ Means ± SE (n = 8/group). ^ns^: There were no significant differences between columns (*p* < 0.05) in Duncan’s multiple range tests after one-way ANOVA. ^3^ FER: Food efficiency ratio = (weight (g)/food intake (g)) × 100.

**Table 3:** Effects of fermentation of sweet red gamju on the gene expression ratio in the serum of Balb/c mice (n = 8/group).

Treatments ^1^	IL-4	IL-6	IL-10
NC	1.00 ± 0.02 ^b,2^	1.00 ± 0.17 ^b^	1.00 ± 0.28 ^b^
PC	0.52 ± 0.02 ^a^	0.52 ± 0.01 ^a^	0.18 ± 0.04 ^a^
SR	0.89 ± 0.17 ^ab^	0.54 ± 0.03 ^a^	0.13 ± 0.14 ^a^
FSR	0.79 ± 0.15 ^ab^	0.75 ± 0.13 ^ab^	0.16 ± 0.12 ^a^

^1^ FSR: Fermented sweet red gamju-treated group; NC: Negative control group; PC: Positive control group; SR: Sweet red gamju-treated group. ^2^ Data are relative values when NC = 1. Superscript alphabets of data were designed according to the difference at *p* < 0.05.

**Table 4:** Determination of Immunoglobulin A (IgA), Immunoglobulin E (IgE), and Immunoglobulin G (IgG).

Treatemnts ^1^	IgA (μg/mL)	IgE (ng/mL)	IgG (μg/mL)
NC	52.3 ± 5.1 ^2,ns^	64.4 ± 2.2 ^ns^	433.5 ± 13.6 ^a^
PC	69.4 ± 7.9	75.7 ± 4.5	803.3 ± 83.4 ^c^
SR	66.9 ± 7.3	69.6 ± 6.4	412.2 ± 44.8 ^a^
FSR	62.5 ± 4.9	65.9 ± 2.1	502.6 ± 25.8 ^b^

^1^ FSR: Fermented sweet red gamju-treated group; NC: Negative control group; PC: Positive control group; SR: Sweet red gamju-treated group. ^2^ These values are means ± SE (n = 8/group); ^ns^: There were no significant differences between the columns (*p* < 0.05) in Duncan’s multiple range tests after one-way ANOVA. Superscript alphabets of data were designed according to the difference at *p* < 0.05.

## Data Availability

Data sharing not applicable.
